# Electrochemical DNA Sensor Based on Carbon Black—Poly(Methylene Blue)—Poly(Neutral Red) Composite

**DOI:** 10.3390/bios12050329

**Published:** 2022-05-12

**Authors:** Dominica Kappo, Dmitry Shurpik, Pavel Padnya, Ivan Stoikov, Alexey Rogov, Gennady Evtugyn

**Affiliations:** 1 A.M. Butlerov’ Chemistry Institute, Kazan Federal University, 18 Kremlevskaya Street, 420008 Kazan, Russia; almija@mail.ru (D.K.); dnshurpik@mail.ru (D.S.); pavel.padnja@kpfu.ru (P.P.); ivan.stoikov@mail.ru (I.S.); 2 Interdisciplinary Center, Analytical Microscopy, Kazan Federal University, 18 Kremlevskaya Street, 420008 Kazan, Russia; aleksej.rogov@kpfu.ru; 3 Analytical Chemistry Department, Chemical Technology Institute, Ural Federal University, 19 Mira Street, 620002 Ekaterinburg, Russia

**Keywords:** DNA sensor, electropolymerization, Methylene blue, Neutral red, pillar [5]arene, doxorubicin determination, voltametric sensor

## Abstract

The detection of small molecules interacting with DNA is important for the assessment of potential hazards related to the application of rather toxic antitumor drugs, and for distinguishing the factors related to thermal and oxidative DNA damage. In this work, a novel electrochemical DNA sensor has been proposed for the determination of antitumor drugs. For DNA sensor assembling, a glassy carbon electrode was modified with carbon black dispersed in DMF. After that, pillar [5]arene was adsorbed and Methylene blue and Neutral red were consecutively electropolymerized onto the carbon black layer. To increase sensitivity of intercalator detection, DNA was first mixed with water-soluble thiacalixarene bearing quaternary ammonium groups in the substituents at the lower rim. The deposition of the mixture on the electropolymerized dyes made it possible to detect doxorubicin as model intercalator by suppression of the redox activity of the polymerization products. The DNA sensor made it possible to determine 0.5 pM–1.0 nM doxorubicin (limit of detection 0.13 pM) with 20 min of incubation. The DNA sensor was successfully tested on spiked samples of human plasma and doxorubicin medication.

## 1. Introduction

Electrochemical DNA sensors are considered a promising tool for determination of various species in medical diagnostics [1], drug screening [2,3], toxicity assessment [4] and environmental monitoring [5]. The unique properties of DNA as a biorecognition element coupled with modern advances in genetic manipulation offer many opportunities to design biochemical receptors for organic species involved in controlling chemical environments. Meanwhile, the present commercial application of DNA-based recognition in sensor technologies is mostly limited by detection of hybridization events between complementary oligonucleotides on the transducer interface [6,7,8]. The determination of small molecules able to specifically interact with native DNA and affect its biochemical functioning calls for the elaboration of certain approaches that consider the difference in the scale of a receptor and an analyte species and relatively minor changes at the sensor interface resulting from their interaction.

Electrochemical principles of biosensor signal transduction offer many advantages related to rather simple design of both the biosensor and measurement equipment, sufficient sensitivity of the signal, well-elaborated theory and intuitively understandable response, easily recorded with conventional measurement equipment [9,10]. Electrochemical biosensors can be easily miniaturized and are frequently used in point-of-care mode [11]. Electrochemical detection of DNA sensor signals is mainly based on the following approaches [12,13,14]: (1) Direct electrochemistry of DNA immobilized on the electrode interface. As an example, the signals of guanine and adenine oxidation frequently coupled with those attributed to their oxidation products are measured with differential pulse voltammetry; (2) Application of covalently attached labels exerting high redox signal in close proximity to the electrode; and (3) Application of diffusionally free redox indicators. Their transfer to the electrode is affected by specific interactions of DNA with analyte molecules. In the latter case, the influence of the steric factors can be extended by electrostatic repulsion/attraction of the charged redox probes. Methylene blue (MB) [15], Ru(II) complexes [16] are examples of such free redox indicators able to intercalate the DNA duplex [17,18].

Label-free protocols of the signal measurement have some undisputable advantages, e.g., no need for chemical modification of the DNA probes implemented in the biosensor assembly. Nevertheless, they assume several steps of reagent addition/biosensor washing and can be complicated by partial losses of surface components or non-specific adsorption of matrix components on the electrode interface.

The use of redox polymers as signal-forming parts of the DNA sensor design is a promising alternative to both label and indicator-based techniques due to multiple electrostatic interactions with phosphate residues of the DNA backbone and high sensitivity of redox characteristics to the DNA microenvironment and its specific reactions [19]. Polyaniline has mostly been described as a DNA carrier, electron transduction amplifier [20,21,22] and signal-forming element in DNA sensors. Its operation is limited by pH sensitivity of redox activity and electroconductivity and the necessity of acidic media for signal transduction.

Phenothiazine and diazine dyes have been also used for electropolymerization and detection of DNA interactions [23,24,25,26]. They showed a wider pH range of operation and sensitivity toward DNA intercalators and DNA-damaging factors. DNA contact with reactive oxygen species or intercalators, both prior to implementation in the biosensor assembly or already placed onto the polymer layer, resulted in shift in redox equilibria of electropolymerized products. Appropriate changes could be monitored by the shift in the peak potentials and peak currents on direct current voltammograms or by variation in the charge transfer resistance in electrochemical impedance spectroscopy [25,26].

Although polyphenothiazines are compatible with DNA and show reproducible signals, their electropolymerization is sometimes less effective than that of aniline and results in rather modest accumulation of the redox-active products on the electrode. A thin redox layer offers higher requirements regarding the reproducibility of the surface morphology and DNA adsorption conditions, and is sensitive to undesirable aggregation of the reactants caused by low solubility of the monomeric and polymeric forms of dyes and their adducts with DNA. This makes the variation range of the signals narrower and the measurements of the DNA-affecting factors semi-quantitative.

The use of macrocyclic compounds bearing functional groups able to bind DNA and participate in electron transduction extends the performance of appropriate DNA sensors. Recently, we have shown the advantages of such an approach on an example of DNA sensors utilizing electropolymerized layers of thionine and Azure B [27]. Introduction of pillar [5]arene (P [5]A) amplified the influence of the redox-active species on the signal of the DNA sensor. Aminated thiacalixarene significantly improved the performance of the DNA sensor when used for doxorubicin determination [28]. The macrocycle promoted the formation of polyelectrolyte complexes between DNA and electropolymerized Neutral red (NR) or polyaniline, and increased sensitivity of the detection of DNA–drug interactions. In both cases, spatial pre-arrangement of the functional groups of the macrocycles provided a more regular structure of the surface layer formed after adsorption of the DNA molecules. The closer position of the charged groups and redox sites resulted in a higher response and possibility to discriminate various factors affecting DNA configuration in the biosensor assembly (intercalation, oxidative and thermal damage).

Many antitumor drugs intercalate the DNA molecules of cancer cells to prevent their transcription and cell division. Intercalation involves insertion of a planar drug molecule between the DNA base pairs followed by distortion of the DNA helix and an increase in DNA volume [29]. Furthermore, anthracycline intercalators promote oxidative DNA damage by stimulation of reactive oxygen species formation and inhibition of DNA reparation systems [30,31]. In spite of their high efficiency, anthracyclines are toxic and show a narrow gap between therapeutic and toxic doses. Progress in the development of new methods of their detection offers safer application protocols and possibilities for screening less-toxic pharmaceuticals. With such methods, it is important to distinguish between the intercalation and DNA-damaging factors (chemical or thermal denaturation of DNA) because they exert similar changes upon the DNA structure, detected by electrochemical instrumentation.

Taking into account the advantages of the use of electropolymerized redox-active materials for electron transduction and of the charged macrocycles for the DNA incorporation, in this work we have studied DNA sensors based on the hybrid poly(MB) and poly(NR) layers polymerized in the presence of P [5]A and DNA introduced in the layer in the complex with cationic water soluble thiacalix [4]arene with terminal quaternary ammonium groups of the substituents at the lower rim. The influence of the thiacalix [4]arene on the introduction of DNA and detection of specific DNA interactions has been assessed.

## 2. Materials and Methods

### 2.1. Reagents

Low-molecular DNA from salmon tests (lyophilized powder, <5% protein, A_260/280_ 1.4, G-C content 41.2%, about 2000 base pairs per molecule), doxorubicin hydrochloride ((7S,9S)-7-[(2R,4S,5S,6S)-4-amino-5-hydroxy-6-methyloxan-2-yl]oxy-6,9,11-trihydroxy-9-(2-hydroxyacetyl)-4-methoxy-8,10-dihydro-7H-tetracene-5,12-dione, 98–102%), MB ([7-(dimethylamino)phenothiazin-3-ylidene]-dimethylazanium chloride, 95%), NR (3-amino-7-dimethylamino-2-methylphenazinium hydrochloride, 90%), potassium hexacyanoferrate (III) (99%), potassium hexacyanoferrate (II) (98.5–102%), poly(sodium 4-styrenesulfonate), mol. weight 70,000 (PSS), sulfamethoxazole, kanamycin and paracetamol were purchased from Sigma-Aldrich, Dortmund, Germany. Doxorubicin TEVA^®^ was purchased in the local drug store. Chitosan (mol. weight 30,000–100,000 D) was purchased from Acros Organics BVBA, Geel, Belgium, and carbon black (CB, >99.95% C) from Imerys Graphite&Carbon, Willebroek, Belgium. All the working solutions were prepared using Millipore Q^®^ water (Simplicity^®^ water purification system, Merck-Millipore, Molsheim, France). Other reagents were of analytical grade. Electrochemical measurements were performed in 0.1 M HEPES containing 0.1 M Na_2_SO_4_, pH = 6.0.

### 2.2. Synthesis of Thiacalix [4]arene Derivatives Bearing Ammonia Groups

**Figure 1:**
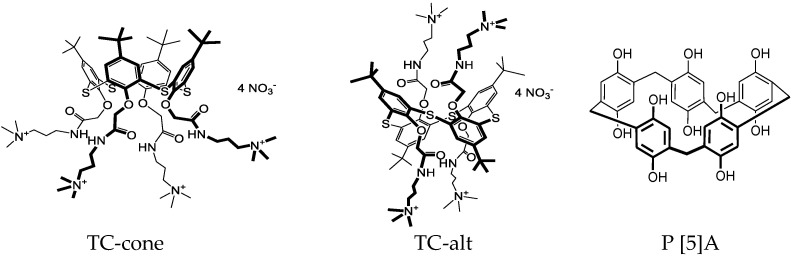
Chemical structures of the macrocycles used in the DNA sensor assembling.

Macrocyclic compounds used in the biosensor assembly were synthesized at the Organic Chemistry Department of Kazan Federal University, as described elsewhere [32,33]. Unsubstituted decahydroxylated P [5]A was obtained by the Ogoshi method [32]. Water-soluble amphiphilic derivatives of thiacalix [4]arene (5,11,17,23-tetra-*tert*-butyl-25,26,27,28-tetrakis-[(*N*-(3′,3′,3′-trimethyl-ammoniumpropyl)carbamoylmethoxy]-2,8,14,20-thiacalix [4]arene tetranitrate) in *cone* and *1,3-alternate* configurations were synthesized from appropriate macrocycles tetrasubstituted at the lower rim with tertiary amino groups, as described in [33]. Their alkylation was performed in acetonitrile under reflux. The yield of target compounds was equal to 90–98%. The structure and purity of the macrocycles synthesized were confirmed by ^1^H NMR and ^13^C NMR spectroscopy, MALDI mass-spectrometry, and elemental analysis. Finally, iodide counter ion was replaced with nitrate by ion-exchange column chromatography. The reaction was monitored using AgNO_3_ reaction with iodide ions. Structural formulae of the compounds are presented in Figure 1.

### 2.3. Electrochemical Instrumentation

Voltammetric and impedimetric measurements were performed at ambient temperature using CHI 660E instrumentation (CH Instruments Inc., Austin, TX, USA). The DNA sensor was assembled on the glassy carbon electrode (GCE, ALS Co. Ltd., Tokyo, Japan, working area 0.283 cm^2^). Pt wire (ALS Co. Ltd., Tokyo, Japan) served as an auxiliary electrode and the Ag/AgCl/1.0 M KCl (CHI 129, CH Instruments) as a reference electrode.

### 2.4. Scanning Electron Microscopy

Scanning electron microscopy (SEM) images of the electrode coatings were obtained with the high-resolution field emission scanning electron microscope Merlin™ (Carl Zeiss, Jena, Germany).

### 2.5. Modification of the Working Electrode

GCE was mechanically polished and washed with acetone, ethanol, and deionized water. After that, it was electrochemically cleaned by repeated cycling the potential in a range from −1.0 to 1.0 V at a scan rate of 100 mV/s in 0.2 M sulfuric acid until stabilization of the cyclic voltammograms (normally within 10 min). Two types of CB suspension were prepared for this purpose. In the first case, 1.3 mg/mL CB dispersion in 0.375% chitosan solution was prepared using 0.05 M HCl. After deposition on the electrode, it was additionally treated with 1 μL of 1.0 M NaOH. In the second case, 1 mg of CB was dispersed in 1 mL of the 1:3 (*v/v*) mixture of concentrated nitric and sulfuric acids and sonicated for 60 min. Then, sediment was isolated by ultracentrifugation, washed with deionized water, and dispersed under ultrasonication in dimethylformamide (DMF). The resulting layer on the electrode was dried at 60 °C. After the CB deposition, 2 μL of 0.2 mM P [5]A solution in acetone was dispersed on the CB layer and left to dry at room temperature. Finally, the electrode was washed several times with deionized water.

### 2.6. Electropolymerization of MB and NR

Both dyes were electropolymerized by repeated cycling of the potential of their solutions in the conditions established earlier in our works on electrochemical DNA sensors [24,25,27,28]. MB was polymerized from its 2.5 mM solution in phosphate buffer containing 0.1 M Na_2_SO_4_, at pH 7.0 and with a potential ranging from −0.6 to 1.2 V (15 cycles). NR was polymerized from its 0.4 mM solution in HEPES containing 0.1 M NaNO_3_ at pH 6.0 and in a potential ranging from −0.80 to 0.80 V (9.5 cycles). The efficiency of electropolymerization was monitored by changes in the peak currents on voltammograms. After electropolymerization, the electrode was transferred to the phosphate buffer with no monomers and stabilized by repeated scanning of the potential in the range applied on the electropolymerization stage until stabilization of the peaks on voltammograms (about 10 min).

### 2.7. DNA Biosensor Assembling and Signal Measurements

The immobilization of DNA was performed by its adsorption on the layer of electropolymerized dyes followed by incubation of the electrode for 60 min in conditions that prevented drying of the solution (under the plastic tube). Electrostatic interactions between negatively charged phosphate residues of the DNA molecule and positively charged polymer chains promoted adsorption. For this reason, the NR polymerization was finished at a maximal positive potential that corresponded to the maximal accumulation of oxidized (positively charged) monomer units of the dye. After that, the electrode was washed several times with deionized water and then working buffer. In some experiments, DNA was mixed with water-soluble thiacalix [4]arenes bearing terminal quaternary ammonium groups at the substituents at the lower rim. The electrode was incubated in the mixture, as described above for DNA solution. Finally, the DNA sensor was dried at ambient temperature on air and used for voltametric measurements. If necessary, DNA sensors packed in alumina foil were stored in dry conditions at 4 °C.

In voltametric experiments, changes in the anodic NR peak currents recorded in direct-current mode were used as a biosensor signal for quantification of the DNA-specific interactions with doxorubicin as intercalator. Matrix effect assessment for doxorubicin determination was performed using artificial plasma containing 4.0 μM methionine, 2.0 mM NaCl, 0.2 mM NaHCO_3_, 1.3 μM L-cysteine, 3.5 μM L-glycine, 0.21 mM L-tryptophan, 0.2 mM L-tyrosine, 4.0 μM L-phenylalanine, 5.0 μM DL-lysine, 3.5 μM L-histidine, 22 μM L-asparagine, 5.0 μM L-arginine, and 0.2 mM L-alanine [31,34].

## 3. Results

### 3.1. Electrochemical Characterization of the Electrode Modification

Electropolymerization of the MB and NR on the GCE modified with CB and P [5]A has been described elsewhere [35]. In this work, polymeric NR was coupled with monomeric MB to enhance the response toward DNA-damaging factors and DNA intercalators. However, leaching MB from the surface layer limited the applicability of the response. In this work, polymerized MB was used to promote the electron exchange to NR and to amplify changes attributed to the DNA interactions on the polymer surface.

**Figure 2:**
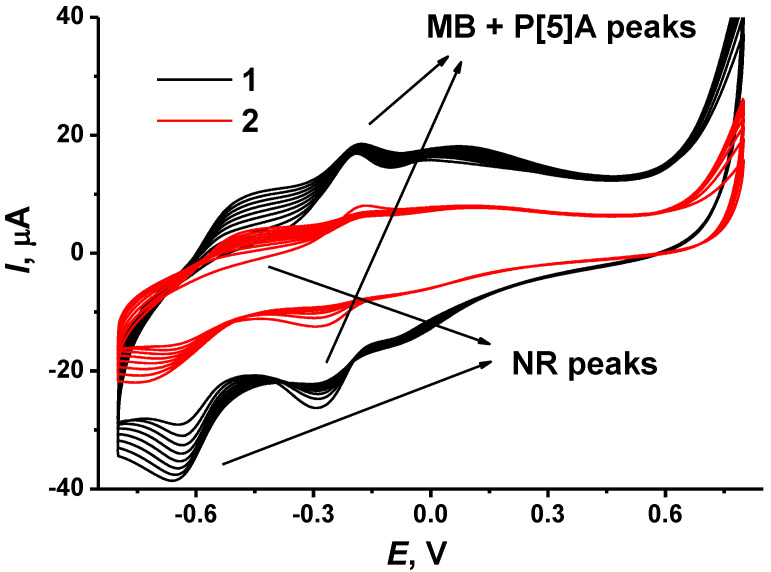
Cyclic voltammograms recorded on GCE covered with P [5]A dispersed in DMF (1) and chitosan (2) and electropolymerized MB in the solution of 0.4 mM NR in 0.1 M HEPES + 0.1 M NaNO_3_, pH 6.0, scan rate 50 mV/s.

It was shown that repeated cycling of the potential resulted in consecutive growth of the peaks attributed to the redox conversion of the dyes and the decrease in the peaks of P [5]A oxidation–reduction. This resulted from the accumulation of the electropolymerization products and simultaneously from partial shielding of the P [5]A molecules in the surface layer. The number of potential cycles was specified from our previous research to establish the most reproducible electrochemical characteristics of the polymer coatings. In case of the NR, potential cycling was finished at the maximum anodic potential to reach a positive charge of the oxidized form of the polymer and to promote adsorption of negatively charged DNA molecules. Consecutive deposition of polymeric dyes showed better results when poly(MB) was obtained first, and poly(NR) was synthesized onto the MB polymerization product. In the opposite order of deposition, the MB peaks dominated, and no influence of NR could be found. For both dyes, the electropolymerization was initiated by primary oxidation of a monomer at high anodic potentials, which corresponded to irreversible peak at 0.75–0.90 V. The efficiency of electropolymerization depended on the CB film-forming agent. It was higher for the CB dispersed in DMF that in chitosan solution. This might result in the formation of a chitosan film on the carbon surface and partial shielding of the CB particles on the electrode interface. Figure 2 depicts electropolymerization of the NR onto the poly(MB)layer. In the following experiments, DMF was used as a film-forming agent for the dispersion and deposition of CB on the GCE.

The growing peak pair at −0.66 …−0.50 V corresponds to the poly(NR). For this polymer, the peaks of the monomer and polymer are observed at the same potentials so that no changes in the peak shape were observed. However, the peak potential difference increased with the number of potential cycles, indicating a growing limitation of the monomer transfer to the electrode interface. The poly(MB) peaks at −0.29 … −0.18 V slightly decreased with NR polymerization. P [5]A did not show its own peaks on the voltammogram; they disappeared with MB polymerization on the preliminary step of the electrode modification (not shown).

The following adsorption of the DNA (1 mg/mL) slightly decreased the peaks of the dyes on the cyclic voltammograms. This was explained by the balance between passive limitation of the electron exchange in the surface layer and electrostatic stabilization of the oxidized state of the polymer layer. Such a mechanism was previously proposed for the interactions of DNA with aminated thiacalix [4]arenes detected on an electrode with methylene green as redox probe [33,36].

**Figure 3:**
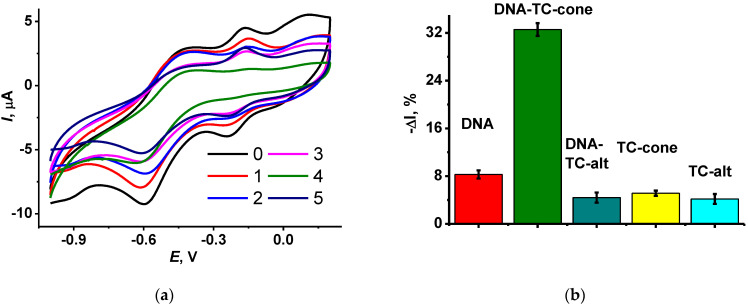
(**a**) Cyclic voltammograms recorded on GCE covered with CB with adsorbed P [5]A and poly(MB)-poly(NR) (0) and that with adsorbed TC-cone (1 mg/mL) (1), DNA (1 mg/mL) (2), DNA (1 mg/mL) + TC-cone (0.5 mg/mL) (3), DNA (1 mg/mL) + TC-cone (1.0 mg/mL) (4), and DNA (1 mg/mL) + TC-cone (2 mg/mL) (5); (**b**) Relative decay of the NR oxidation peak current after deposition of DNA (1 mg/mL) and/or TC (1 mg/mL). Measurements in 0.1 M HEPES + 0.1 M NaNO_3_, pH 6.0.

The addition of TC bearing terminal ammonium groups (see structures in Figure 1) to the DNA solution suppressed the redox activity of the polymer layer. The macrocycle formed an adduct with the DNA molecules, where cationic ammonium groups were located near negatively charged phosphate residues of the DNA backbone. Lower electrostatic interaction with the oxidized NR units of the polymer resulted in shifting the equilibrium of the electron exchange toward reduced forms of the dyes. Hence, the peak currents became lower. Figure 3 displays the relative decrease in the NR oxidation peak current for various Macrocycles added to the modified electrode. The effect is much less pronounced for the TC–alt. It is likely that the *1,3-alternate* configuration of the macrocycle does not provide necessary conformance of the charge distribution of the reactants, or the equilibrium of the adduct formation is shifted to the initial substance while being transferred on the electrode interface. It should be also noted that the macrocycles themselves also affect the peak currents but to a lower extent. The maximal shift in the NR oxidation peak current of 32% was reached for 1 mg/mL DNA and TC-cone. The following increase in the macrocycle concentration did not significantly change this level, but the deviation of the response increased from 3–4% for the smaller macrocycle quantities to 7–8% for the 2 mg/mL TC-cone. This might be caused by low solubility of the TC macrocycles or their aggregation in the aqueous solution.

The electrostatic nature of the effect of TC macrocycles on the DNA sensor response was confirmed using PSS instead of DNA. Similar changes in the NR peaks on voltammograms were observed, with a maximal decay of 15% within the same range of the polyelectrolyte concentrations.

Following the results obtained with two TC configurations, the electrochemical characterization and assembling of the DNA sensor was performed using only the TC-cone macrocycle.

Coatings obtained were characterized using variations in the scan rate (ν) of the potential and the NR peak currents. The pH dependence on the NR peak potential was within a range from 3.0 to 8.0. The results obtained are summarized in Table 1. As can be seen, the electron transfer is limited from the formal point of view by surface-confined reaction and diffusion. In this case, all the redox species are located on the electrode interface and the diffusion is replaced by electron exchange within the polymer layer. The slopes of the anodic and cathodic peak current (*I_p_*) dependencies are similar to each other. They slightly decrease with the implementation of the macrocycle and return to initial values when the TC-cone–DNA adduct was adsorbed on the electrode. The intercept of the log*I_p_*–log v plots increased with the quantities of electrochemically inactive components, indicating a growing contribution of the capacitance currents.

**Figure 4:**
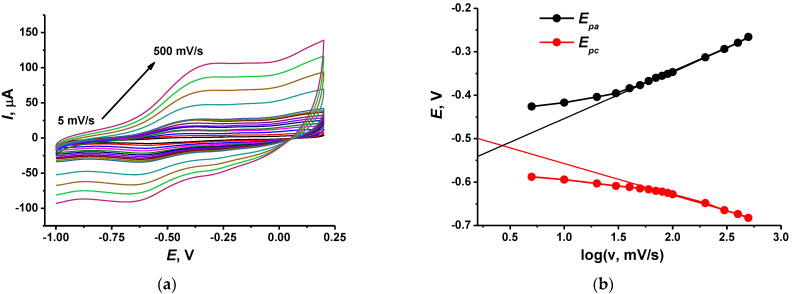
(**a**) Cyclic voltammograms recorded on the GCE covered with CB, P [5]A, poly(MB), poly(NR) and adsorbed DNA (1 mg/mL)–TC-cone (1 mg/mL) adduct. Scan rate range 5–500 mV/s; (**b**) Determination of the electron transfer coefficients from the *E*–logν dependence.

The electron transfer coefficient was calculated from the slope of the linear approximation of the *E_p_*—logv dependency at high scan rates [37] in accordance with Equation (1). Figure 4 shows appropriate graphs for the electrode containing DNA–TC-cone adduct.

(1) $$\alpha_{c}=-\frac{2.303RT}{F}\frac{d\log\left|I_{pc}\right|}{dE};\;\alpha_{a}=\frac{2.303RT}{F}\frac{d\log\left|I_{pa}\right|}{dE}$$

Here, *α_c_* and *α_a_* are cathodic and anodic transfer coefficients, and *F*, *R*, and *T* have their usual significance. One electron transfer in the limiting step is assumed.

Deposition of TC-cone and DNA, alone or together, did not significantly alter the *α_a_* value, which was about 0.69–0.72. Meanwhile the cathodic transfer coefficient increased from 0.30 for the TC-cone layer to 0.41 for the DNA–TC-cone adduct (all the concentrations equal to 1.0 mg/mL). This coincides well with the influence of the macrocycle on the general charge separation on the electrode interface and its ability to partially shield the negative charge of the DNA molecules.

The pH dependence of the potential mostly corresponds to the transfer of two electrons and one hydrogen ion in oxidation path. Regarding the reduction step, an equal number of electrons and H^+^ ions is transferred in acidic media, and two electrons and one hydrogen ion in basic media. This stoichiometry coincides with that reported for NR electropolymerization in the literature [38].

### 3.2. SEM Measurements

**Figure 5:**
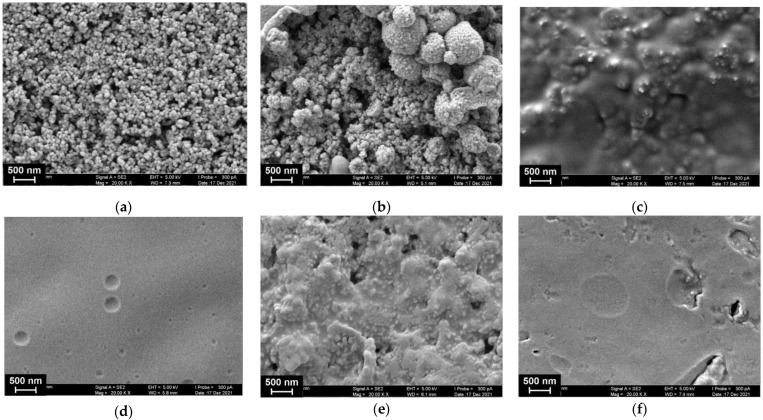
SEM images of the GCE surface consecutively modified with the following components: CB (1 mg/mL dispersion in DMF) and P [5]A (2 μL of 0.2 mM solution in acetone per electrode) (**a**) poly(MB) (**b**), poly(NR) (**c**), DNA (1 mg/mL) (**d**), DNA (1 mg/mL) + TC-cone (1 mg/mL) (**e**). (**f**) Shows that the morphology of the surface layer consisted of the polymerized dyes and TC-cone adsorbed on the electrode with no DNA.

The redox activity of the hybrid poly(MB)–poly(NR) coating was similar to that of the [Fe(CN)_6_]^3−/4−^ redox probe. This did not allow for the use of electrochemical impedance spectroscopy to confirm the surface layer formation. Instead, the morphology of the electrode working area was monitored with SEM (Figure 5).

As can be seen from Figure 5a, P [5]A molecules are preferably adsorbed on the CB particles that preserve their roundish shape and regular distribution on the GCE surface. The electropolymerization of MB results in the formation of bigger particles that are placed on the CB layer but do not fully cover them. The following deposition of the NR resulted in a full coverage of the surface with a dense film that leaves the relief of underlying particles. The following adsorption of DNA resulted in formation of a uniform film. This might result from the electrostatic interactions because the electropolymerization of the NR films finished at maximal anodic potential, and hence resulted in the formation of the maximal number of oxidized dye units nearing a positive charge. It should be also noted that the formation of such a film did not prevent the redox reactions in the underlying layers of the dyes, so changes in the peak currents on the voltammograms were lower than could be expected for diffusionally free redox probes in the same conditions. The reaction of DNA with TC-cone bearing quaternary ammonium groups promoted aggregation of the adducts on the electrode and partial liberation of the grained structure. Deposition of the TC-cone with no DNA resulted in the formation of a film with numerous raptures and defects, probably due to hydrophobic interactions.

### 3.3. DNA and Doxorubicin Influence

As stated in preliminary experiments, exposure of the GCE covered with poly(MB) and poly(NR) composite decreased the redox peaks on voltammograms attributed to the polymeric dyes. In the presence of TC-cone, the DNA influence was increased four-fold. This effect is more pronounced for the NR oxidation peak current, which was used in the following experiments as a measure of DNA-related interactions in the surface layer. The influence increased both with the concentration of the macrocycle (see Figure 3a) and DNA. In the latter case, saturation was observed at 2 mg/mL DNA. The following increase in the DNA concentration resulted in a higher deviation of the peak current due to irregular deposition of the biopolymer on the underlying polymeric film.

**Figure 6:**
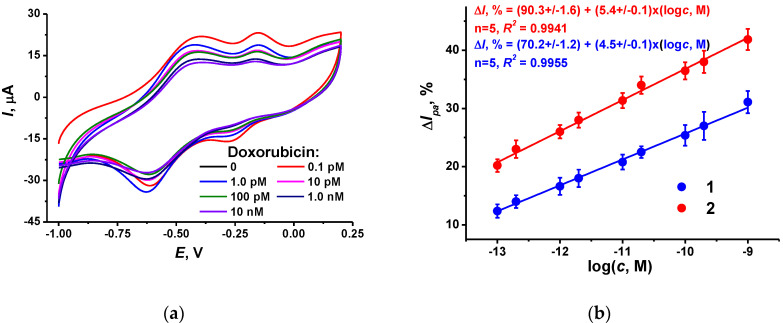
(**a**) Cyclic voltammograms recorded on the GCE covered with CB, P [5]A, poly(MB), poly(NR) and adsorbed DNA (1 mg/mL)–TC-cone (1 mg/mL) after incubation in 0.1, 1.0 pM, 0.01, 0.1 and 1.0 nM doxorubicin; (**b**) Calibration plots of doxorubicin obtained with the DNA sensors utilizing the CB dispersion in chitosan (1) and DMF (2). Incubation 20 min.

Incubation of the DNA sensor in doxorubicin solution resulted in a further decrease in the NR peak current. This coincides with the intercalation of the drug into the DNA helix followed by changes in the charge distribution and DNA–TC-cone interactions. This might result in the formation of a denser layer, preventing the electron exchange in the polymeric dyes. Appropriate voltammograms and calibration curves are presented in Figure 6. The effect of doxorubicin increased with incubation time until 20 min. All the following experiments were performed within this incubation period.

For comparison, appropriate dependence obtained with the CB dispersed in chitosan has been presented in Figure 6b (line 1). The slope of linear approximation is rather close to that obtained with the CB layer dispersed in DMF (line 2). This confirms the suggestion about partial shielding of the CB particles in the chitosan layer expressed after the investigation of the dye electropolymerization efficiency (Figure 1). The slope of the doxorubicin calibration curve in the semi-logarithmic plots is about 5–6% per decade, or twice higher than the doubled deviation of the peak current shift measured. The limit of detection (LOD) was calculated as a doxorubicin concentration corresponding to a 15% decay of the peak current. It was found to be 0.13 pM for the DNA sensor based on CB dispersion in DMF. This is comparable to or better than the performance of voltametric and DNA sensors described in the literature (Table 2).

It is interesting to compare the performance of the DNA sensors utilizing thiacalixarenes [28,32,33,35,36]. In these, terminal amino groups were introduced to the substituents at the lower rim of the macrocycle. The greatest difference in DNA sensor assembly was seen in this work. Here, the DNA was first mixed with the TC-cone and placed on the electrode surface, while in other biosensors, macrocycles were first deposited onto the GCE or electropolymerized film and then the DNA aliquot was added so that the reaction of the components was limited by heterogeneous conditions and steric hindrance of adduct formation. Second, aminated thiacalixarenes are less adapted for the reaction with anionic sites of DNA against TC-cone, with quaternary ammonium groups separated from the macrocycle core with rather flexible linkers. For this reason, the formation of the DNA–macrocycle adducts is more sensitive to electrostatic interactions on the biosensor interface and reacts even to minor changes in the charge distribution resulting from doxorubicin intercalation. Overall changes in the currents are not very high and cover about 30–40% of the initial peak current. Nevertheless, they are quite significant and applicable for semi-quantitative assessment of the intercalating drugs in various media.

It should be noted that the signal of the DNA sensor developed was stable after contact with some other drugs, e.g., sulfamethoxazole, kanamycin and paracetamol, in a micromolar concentration range. The choice of sulfamethoxazole as a representative of sulfanilamide medications is explained by common treatment of patients with such antibiotics to prevent hospital-acquired infections. Furthermore, a comparison of the influence of anthracycline and sulfanilamide drugs has been previously performed for polyaniline–DNA composites [47]. Kanamycin is an aminoglycoside antibiotic that can affect the charge of the surface layer and hence interfere with doxorubicin determination. This is due to aminated groups in its structure that are able to perform electrostatic interactions with charge functions of the layer. Paracetamol is one of the most frequently used medications for treating fever and relieving pain. It exerts electrochemical activity and is often considered as a potential interference in electrochemical methods of drug determination. In this study, its application did not alter the signal toward doxorubicin due to the low potential of the NR peaks used as signals to doxorubicin. Meanwhile, it is expected that the DNA sensor based on the intercalation effect does not allow for discriminating anthracycline drugs (doxorubicin, daunorubicin, idarubicin), which likely affect the signal in a similar manner. Previously, this was shown for another DNA sensor based on polyaniline as a signal-forming component of the sensing layer [47]. This coincides with common clinical practice of the application of individual anthracycline with cardio protectors, often in liposomes [48].

### 3.4. Measurement Precision and DNA Sensor Lifetime

Sensor-to-sensor repeatability was calculated from the results of voltametric measurements performed with six individual DNA sensors incubated in 0.1 nM doxorubicin for 20 min. The GCE covered with the copolymer of the Azure B and proflavine can be stored in dry conditions at 4 °C for at least six months. In the following DNA application and doxorubicin signal measurement, the deviation tends to increase to 10% toward the end of the storage period.

### 3.5. Real Sample Analysis

**Figure 7:**
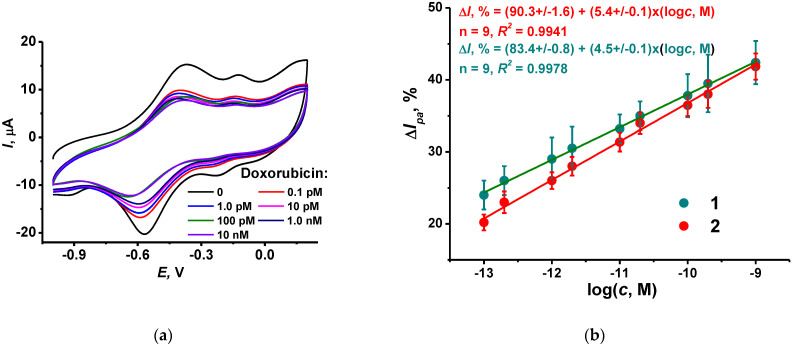
(**a**) Cyclic voltammograms recorded on the GCE covered with CB, P [5]A, poly(MB), poly(NR) and adsorbed DNA (1 mg/mL)–TC-cone (1 mg/mL) after incubation in spiked plasma containing 0, 0.1, 1.0 pM, 0.01, 0.1 and 1.0 nM doxorubicin; (**b**) Calibration plots of doxorubicin obtained with the DNA sensor in spiked plasma (1) and standard doxorubicin solutions in 0.1 M HEPES, pH 6.0 (2). Incubation 20 min.

To show the applicability of the DNA sensor, it was applied for the assessment of the doxorubicin residues in spiked samples of synthetic human plasma (see content in Experimental section). Each DNA sensor was used once because of random changes in the response observed for its repeated incubation in spiked plasma. These can be attributed to incomplete removal of the species interacting with DNA in the intermediate washing step or deposition of colloidal particles present in real samples and drug medications. Prior to incubation, the pH of the spiked plasma was measured and corrected to pH = 7.0 if necessary. The resulting changes in the poly(NR) peak shape and height were similar to those obtained in HEPES buffer (Figure 7).

The DNA sensor was also used for the determination of doxorubicin in Doxorubicin-TEVA ^®^ medication (lyophilizate for injection solutions). It was dissolved in 0.1 M HEPES to a nominal concentration of 1.0 nM and then used for the signal measurement, as described above. The recovery was assessed to be 95 ± 8% (three measurements). No influence of stabilizers present in the medication (lactose) was found.

## 4. Discussion

The results obtained indicated the importance of the assembling the DNA sensor for sensitive detection of biospecific interactions. In this work, DNA was first mixed with the macrocycle bearing quaternary ammonium groups at the substituents at the lower rim of the thiacalixarene core. The compliance of the binding sites was reached only for the *cone* configuration. The underlying layer of the biosensor consisted of CB particles dispersed in the DMF as a film-forming agent, and of the electropolymerized MB and NR dyes. Although all the components of the layer have been tested previously, their combination resulted in a remarkable increase in the sensitivity of the DNA sensor toward the model intercalator (doxorubicin). Thus, the maximal relative shift of the signal (anodic peak current attributed to the poly(NR)) was 1.5 times bigger than that earlier obtained with no thiacalixarene [32]. As a result, the LOD achieved for doxorubicin was decreased by about two orders of magnitude. The mechanism of such changes in the redox behavior of the polymerized dye assumes that the formation of the DNA–thiacalixarene adduct affected the equilibrium of the redox conversion of the NR fragments. Doxorubicin intercalation influenced both the charge distribution and the volume of the biopolymer. Further, it could promote changes in the aggregation of the reactants on the electrode interface. This latter statement was proved using SEM for the assessment the morphology of the surface layer.

The DNA sensor developed showed stable and reproducible signals both in model solutions of the intercalator and in spiked plasma. No influence of several other antibiotics on the signal was found. This offers good opportunities for the application of such DNA sensors in pharmacokinetics of anthracycline preparations and their manufacture control.

## Figures and Tables

**Table 1:** The dependence of the NR peak currents on the scan rate and peak potential on the pH for GCE covered with the CB, P [5]A, redox polymers and DNA. Average ± S.D. from six measurements.

Surface Layer	NR Oxidation Peak			NR Reduction Peak		
	a ± Δa	b ± Δb	*R* ^2^	a ± Δa	b ± Δb	*R* ^2^
Regression Equation: log(*I_p_*, μA) = a + b × log(ν, V/s)						
Poly(MB)-poly(NR)	0.016 ± 0.029	0.774 ± 0.014	0.9959	0.153 ± 0.029	0.758 ± 0.015	0.9955
Poly(MB)-poly(NR)-TC-cone	0.056 ± 0.034	0.696 ± 0.018	0.9906	−0.041 ± 0.004	0.715 ± 0.019	0.9939
Poly(MB)-poly(NR)-TC-cone-DNA	−0.208 ± 0.018	0.783 ± 0.009	0.9984	−0.298 ± 0.026	0.826 ± 0.013	0.9975
Regression equation: *E*, mV = a + b × pH						
Poly(MB)-poly(NR), pH = 3–5	−0.157 ± 0.008	−0.038 ± 0.001	0.9929	−0.144 ± 0.017	−0.085 ± 0.004	0.9953
pH = 5–8				−0.369 ± 0.021	−0.041 ± 0.003	0.9816
Poly(MB)-poly(NR)-TC-cone, pH = 3–5	−0.123 ± 0.026	−0.048 ± 0.006	0.9652	−0.144 ± 0.016	−0.093 ± 0.004	0.9961
Poly(MB)-poly(NR)-TC-cone, pH = 6–8	−0.097 ± 0.060	−0.048 ± 0.009	0.9373	−0.387 ± 0.033	−0.041 ± 0.005	0.9728
Poly(MB)-poly(NR)-TC-cone-DNA, pH = 3–5	−0.124 ± 0.023	−0.048 ± 0.006	0.9710	−0.176 ± 0.008	−0.084 ± 0.002	0.9990
Poly(MB)-poly(NR)-TC-cone-DNA, pH = 6–8	−0.150 ± 0.062	−0.030 ± 0.009	0.9061	−0.350 ± 0.011	−0.045 ± 0.002	0.9975

**Table 2:** The comparison of the performance of voltametric sensors and biosensors for doxorubicin determination.

Surface Layer Content	Concentration Range	LOD, nM	Refs.
Voltametric Sensors			
Graphene, poly(taurine), β-cyclodextrin	6 nM–3.45 μM	12	[39]
Ag nanoparticles, chitosan	0.103–3.6 μM	103	[40]
CB, Cu nanoparticles, Nafion	0.45–5.1 μM	24	[41]
Multi-wall carbon nanotubes, mesoporous Pd and Pt particles	4.4 nM–8.58 μM	0.86	[42]
DNA sensors			
Poly(Azure B–proflavine)	0.03–10 nM	0.01	[26]
Polyaniline or poly(NR), aminated thiacalix [4]arene	1 nM–50 μM	0.1	[28]
CB, P [5]A, poly(NR), monomer of MB	10 nM–0.1 mM	3	[35]
CB, chitosan, aminated thiacalix [4]arene	10 pM–1 nM	0.0003	[36]
Pt and Ag nanoparticles	172 pM–1.72 nM	-	[43]
Single-wall carbon nanotubes	1.0 nM–20 μM	0.6	[44]
Multi-wall carbon nanotubes, poly(L-lysine)	2.5 nM–0.25 μM	1.0	[45]
Polyaniline layered films	1.0 pM–1 mM	0.0006	[46]
CB, P [5]A, poly(MB), poly(NR), thiacalix [4]arene with quaternary ammonium terminal groups	0.5 pM–1.0 nM	0.00013	This work

## Data Availability

Not applicable.
